# Explainable machine learning methods and respiratory oscillometry for the diagnosis of respiratory abnormalities in sarcoidosis

**DOI:** 10.1186/s12911-022-02021-2

**Published:** 2022-10-20

**Authors:** Allan Danilo de Lima, Agnaldo J. Lopes, Jorge Luis Machado do Amaral, Pedro Lopes de Melo

**Affiliations:** 1 Electronic Engineering Post-Graduation Program, State University of Rio de Janeiro, Rio de Janeiro, Brazil; 2 Pulmonary Function Laboratory, Faculty of Medical Sciences, State University of Rio de Janeiro, Rio de Janeiro, Brazil; 3 Department of Electronics and Telecommunications Engineering, Rio de Janeiro State University, Rio de Janeiro, Brazil; 4 Biomedical Instrumentation Laboratory, Institute of Biology Roberto Alcantara Gomes and Laboratory of Clinical and Experimental Research in Vascular Biology (BioVasc), Rio de Janeiro State University, Rio de Janeiro, Brazil

**Keywords:** Forced oscillation technique, Clinical decision support system, Respiratory diseases, Machine learning

## Abstract

**Background:**

In this work, we developed many machine learning classifiers to assist in diagnosing respiratory changes associated with sarcoidosis, based on results from the Forced Oscillation Technique (FOT), a non-invasive method used to assess pulmonary mechanics. In addition to accurate results, there is a particular interest in their interpretability and explainability, so we used Genetic Programming since the classification is made with intelligible expressions and we also evaluate the feature importance in different experiments to find the more discriminative features.

**Methodology/principal findings:**

We used genetic programming in its traditional tree form and a grammar-based form. To check if interpretable results are competitive, we compared their performance to K-Nearest Neighbors, Support Vector Machine, AdaBoost, Random Forest, LightGBM, XGBoost, Decision Trees and Logistic Regressor. We also performed experiments with fuzzy features and tested a feature selection technique to bring even more interpretability. The data used to feed the classifiers come from the FOT exams in 72 individuals, of which 25 were healthy, and 47 were diagnosed with sarcoidosis. Among the latter, 24 showed normal conditions by spirometry, and 23 showed respiratory changes. The results achieved high accuracy (AUC > 0.90) in two analyses performed (controls vs. individuals with sarcoidosis and normal spirometry and controls vs. individuals with sarcoidosis and altered spirometry). Genetic Programming and Grammatical Evolution were particularly beneficial because they provide intelligible expressions to make the classification. The observation of which features were selected most frequently also brought explainability to the study of sarcoidosis.

**Conclusions:**

The proposed system may provide decision support for clinicians when they are struggling to give a confirmed clinical diagnosis. Clinicians may reference the prediction results and make better decisions, improving the productivity of pulmonary function services by AI-assisted workflow.

## Introduction

Sarcoidosis is an inflammatory disease characterized by granulomas, which can appear in practically any organ [1], although the lung is the most common site. Over 150 years after its first clinical description, the cause of sarcoidosis remains unknown, and its treatment is generally unsatisfactory [2]. This disease is characterized by respiratory abnormalities associated with increased airway obstruction and reduced pulmonary compliance [3].

Respiratory changes in these patients are usually evaluated using spirometry. However, these tests demand great effort in executing the forced expiratory maneuver, which can cause changes in bronchomotor tone. This negatively affects the quality of the results [4, 5], and renders the obtained indices hardly physiologic [6]. In addition, it requires significant and coordinated inspiratory and expiratory efforts from the patients, and therefore, it is not suitable for people with serious illnesses [7].

Respiratory oscillometry (also known as the forced oscillation technique—FOT) is a non-invasive method that requires only passive patient cooperation [8]. This method allows the evaluation of the mechanical properties of the respiratory system using the concept of respiratory impedance. We can interpret this impedance physiologically through models of pulmonary mechanics, such as the extended Resistance-Inductance-Capacitance (eRIC) model [9]. This procedure allows us to obtain information concerning central and peripheral airways as well as respiratory compliance.

The FOT complements spirometric analysis by providing extra features for analysis, bringing a more detailed assessment [10], which can help diagnose abnormal changes in sarcoidosis and other respiratory diseases. Although there is increasing research on oscillometry and increased interest and feasibility in its clinical application, the benefits of oscillometry in medicine still need to be realized [8]. One of the main obstacles is that interpreting resistance and reactance curves and the features derived from these curves can be challenging tasks requiring training and experience. Thus, a good way is to use Machine Learning (ML) algorithms to generate interpretable results. However, there are still no studies in the literature using interpretable ML methods associated with FOT measurements to improve the diagnosis of respiratory changes associated with sarcoidosis.

In this context, our specific objectives were (1) to assess the ability of each FOT feature to diagnose respiratory changes associated with sarcoidosis properly; (2) to develop classifiers with different methods to achieve high accuracy on that issue; (3) to explore techniques that generate interpretable results and compare their performance with the most accurate methods.

## Methods

The Research Ethics Committee of the Pedro Ernesto University Hospital (HUPE) approved the study that obeys the Declaration of Helsinki. The written post-informed consent of all volunteers was obtained before inclusion in the study.

### Studied subjects

The data used in this work were obtained through the FOT. The examinations were carried out at the Biomedical Instrumentation Laboratory of the Rio de Janeiro State University. The exam with each volunteer was repeated three times, and each piece of data used in this work results from the average of these three measures. Seventy-two individuals took part in the study. Twenty-five were healthy volunteers representing the control group, and 47 were patients with sarcoidosis. In the latter, spirometry verified that 24 had normal conditions, representing the normal spirometry group, and 23 had respiratory changes, representing the altered spirometry group.

### Forced oscillation measurements and features

The FOT comprises applying oscillations with a low-pressure amplitude to an individual's respiratory system using an external device. While the individual remains seated, wearing a nose clip, and breathing spontaneously, pressure signals with frequencies multiple of 2 in the 4-32 Hz range are applied to the respiratory system's entrance. We measured the applied pressure (P) and the airflow (V′) induced by it. Then, the Fourier transform (F) was used to estimate the respiratory impedance (Z_rs_ = F(P)/F(V′), from which we can generate resistance and reactance curves as a function of frequency.

To interpret the resistance data, we used a linear regression in the 4–16 Hz range to estimate resistance at the intercept (R_0_), the slope of this curve (S) and the average resistance in this range (R_m_). R_0_ and S are related to the respiratory system's total resistance and ventilation inhomogeneity, respectively, and R_m_ is related with central airways' resistance [11].

The resistance measured at low frequency is associated with the airways' total resistance, while at high frequency, it is related with the central airways’ resistance. The difference between them is usually interpreted as an index of small airway obstruction and heterogeneity of ventilation [12]. Then, the other features analyzed are the resistance at 4 Hz (R4), the resistance at 20 Hz (R20), and the difference between them (R4–R20).

To interpret the reactive results, we calculated dynamic compliance (C_dyn_) from the reactance obtained at 4 Hz [13]. In this same frequency, we calculated the absolute value of the respiratory impedance (Z4), a feature associated with the respiratory muscles' work to overcome resistive and elastic loads, to allow the airflow in the respiratory system [11]. The average reactance (X_m_) is also associated with the inhomogeneity of the respiratory system, and we calculated it through the reactance curve based on the entire frequency range studied (4–32 Hz) [14]. We also evaluated the resonant frequency (F_r_), where respiratory elastance and inertance make equal and opposite contributions, resulting in a zero value for reactance). Finally, we measured the area under the negative part of the reactance curve (A_x_), between 4 Hz and Fr, which reflects the elastic properties and ventilation heterogeneity of the respiratory system [15].

### Extended RIC model features

**Fig. 1:**
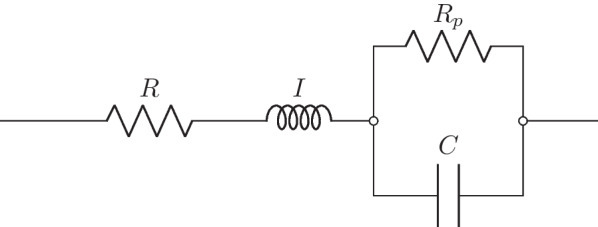
Electrical representation of a two-compartment extended RIC (eRIC) model to analyze respiratory impedance. Resistance, inductance, and capacitance are mechanical resistance, inertance, and compliance analogs, respectively. R is analogous to central airway resistance, and Rp describes peripheral resistance. I is associated with lung inertance and C with respiratory compliance. This analysis also evaluated the total resistance (Rt = R + Rp), which included the effects of central and peripheral airways

The impedance curves provided by FOT may be interpreted using engineering concepts to correlate them with models composed of electrical components analogous to resistance, inertance, and complacency of the respiratory system. The extended RIC (eRIC) model used (Fig. 1) contains a peripheral resistance (R_p_) associated in parallel with the respiratory compliance (C), in series with the central resistance (R) and the respiratory inertance (I) [12]. We define the total resistance (R_t_) as the sum of R and R_p_.

Several studies have already been carried out using this model, such as, associating model features with abnormalities in silicosis [16], showing that the models can aid in the early diagnosis of chronic obstructive pulmonary disease (COPD) [17] and using these features to detect mild obstruction in asthma [18]. We can calculate the impedance equivalent to the eRIC circuit according to Eq. 1.

1 $$Z = R + \frac{{R_{p} }}{{1 + \left( {\omega R_{p} C} \right)^{2} }} + j\left( {\omega I - \frac{{\omega R_{p}^{2} C}}{{1 + \left( {\omega R_{p} C} \right)^{2} }}} \right)$$

Thus, it is necessary to find the values of the features to minimize the error between the impedance measured at discrete frequencies and its respective analytical result. We have estimated using the ModeLIB program developed in our laboratory, which estimated model parameters using the Levenberg–Marquardt algorithm to determine the set of coefficients of the nonlinear model that best represents the input data set in the least-squares sense.

### Datasets

This study carried out the experiments in a dataset with 16 input features (11 FOT indexes and five eRIC model components) from 72 exams. The measurements were performed in 25 healthy volunteers and 47 patients with sarcoidosis: 24 with normal conditions according to the spirometry and 23 with respiratory changes.

### Machine learning algorithms

Machine Learning (ML) is a field of Artificial Intelligence that gives computers the ability to learn without being explicitly programmed to do so [19]. We can use its methodologies mainly in problems with no deterministic solution, using data so that the algorithms automatically discover the relationship between them. Artificial intelligence/machine learning methods have been developed to improve pulmonary function analysis since the 1980s [20]. Previous works have reported that it is workable to use the features obtained by FOT to apply ML algorithms to improve the diagnosis of respiratory diseases [13, 21–25]. Besides providing accurate results, the explanation of a classifier is relevant in the study of respiratory diseases. Knowing how the classification is performed and the most important features can enhance our knowledge about the diagnosis and contribute to our understanding of the underlying pathophysiology. The development of a set of interpretable models and methodologies that result in more understandable models while maintaining excellent prediction performance is the major goal of a new topic of study called Explainable Artificial Intelligence (XAI) [26]. Regrettably, there is no universally accepted definition of explainable. Some researchers use the terms interpretability and explainability interchangeably, while others distinguish between the two. Authors [27] define interpret as “to explain or present in language that humans can understand.” Authors in [28] define interpretation as the translation of abstract concepts into a domain humans can understand, whereas explanation is the collection of the features of the interpretable domain that have led to the production of a choice in a specific example. The notion of explanation and interpretation in this work is aligned with [28].

Therefore, in this study, we want to explore Genetic Programming (GP) because of the classification being made by intelligible expressions that can be interpreted and also study the subset of optimal features selected by the feature selection methods to explain which FOT parameters are most discriminative.

**Fig. 2:**
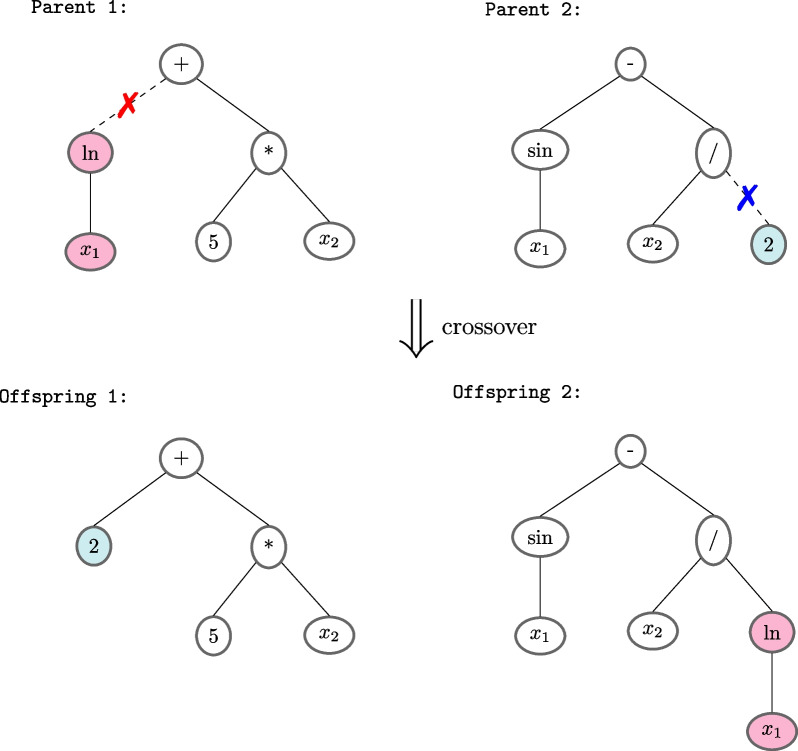
Example of crossover operation in GP individuals

GP is a method used to build programs, which fits into the family of evolutionary algorithms. Each program is an individual whose fitness depends on the execution of that program. The most common representation for a GP individual is as a tree [29]. The terminal nodes (leaves) represent the features, and the internal nodes represent the functions that operate the leaves. Figure 2 shows the tree representation of the program *y* = *ln*(*x*_*1*_) + 5 × *x*_*2*_*.* as parent 1, and the program *y* = *sin*(*x*_*1*_) − *x*_2_/2 as parent 2. However, other forms of representation have become popular, such as graphs, lists, and grammars [30]. In each case, the genotype is the computational representation of the program, and the phenotype is its interpretation, more understandable to the user. Some of the most important characteristics of genetic programming are that it does not require or requires only minimal pre-processing of inputs or post-processing of outputs, and it has a built-in feature selection mechanism that allows GP to select only the more useful features from the dataset. The evolutionary process takes place in the problem domain. Because the outputs are already expressed in this problem domain, there is no need for translation or mapping processes [29].

The proceeding followed by the GP comprises randomly generating the first population and evolving it through generations until a stop criterion is reached, such as, for example, whether we found an optimal individual or we have reached a maximum number of generations. Each generation consists of evaluating each individual's fitness and selecting some of them to apply genetic operators generating offspring. Individuals are chosen on a probabilistic basis based on their aptitude. Individuals with higher fitness, therefore, have a better chance of being chosen. The tournament method is the most commonly used selection method in genetic programming. This method involves selecting a subset of individuals at random from the population. They are compared, and the best individual from this group is chosen to be the parent. In terms of evolutionary operators, genetic programming favors the crossover operator. The subtree crossover operator is the most commonly used crossover operator. A crossing point (node) in two parents is chosen at random and independently in this method. The offspring is formed by removing from the parents the subtrees whose roots are the chosen crossing points. The rest of the trees are combined at these points. Figure 2 shows an example of this process, where the crossing points and the corresponding subtrees are highlighted. Then, parents 1 and 2 are combined to generate offspring 1 and 2. This process is done with copies of the selected parents, thus not eliminating the parents in the process. The most frequently used mutation operator is the subtree mutation. In this operator, a mutation point is chosen randomly and the subtree whose root is the mutation point is replaced by a randomly generated subtree.

The Grammatical Evolution (GE) algorithm [30–32] is based on both the biological process of producing a protein from genetic material and the broader genetic evolutionary process. The genome is composed of DNA that is transcribed into RNA as a string of building blocks. After that, the RNA codons are translated into amino acid sequences and used in the protein. The phenotype is the protein's response to its surroundings. A phenotype is a computer program that is derived from a binary string genome. The genome is decoded into a series of integers that are then mapped onto the program's pre-defined rules, known as grammar, which are defined in Backus–Naur Form (BNF). To map genotype to phenotype, a one-to-many process with a wrapping feature is used. This is analogous to the biological process that occurs in many bacteria, viruses, and mitochondria where the same genetic material is used to express multiple genes. The mapping increases the robustness of the process, both in terms of being able to use structure-agnostic genetic operators on the sub-symbolic representation during the evolutionary process and of being able to generate well-formed executable programs from the representation. Thus, even if the fundamentals are the same, using a different grammar can cause a model to produce significantly different results. This adaptability allows grammar to be applied to a wide range of problems, making it extremely useful.

We used GE and tree-based GP as interpretable classifiers. They can derive a mathematical expression to compute a score that indicates the probability that a patient belongs to a specific class, or they can synthesize Fuzzy Pattern Trees [33].

Because it allows data knowledge to be expressed in a comprehensible form, similar to natural language, fuzzy set theory has provided a framework for developing interpretable models [34, 35], giving the model a higher degree of interpretability. The majority of fuzzy models developed are rule-based fuzzy systems (FBRS), which can represent both classification and regression. It may be difficult to obtain fuzzy models based on easily interpretable rules because, depending on the application, many rules with many antecedents may be required, making the model difficult to understand. A system with fewer rules, on the other hand, is easier to understand, but its predictive accuracy suffers as a result. Therefore, we decided to employ the Fuzzy Pattern Trees (FPT) method, which is based on the theory of fuzzy sets and is not based on rules but on a hierarchical method.

Terminal nodes in FPTs have fuzzy features, and internal nodes have fuzzy operators. FPTs can employ a variety of operators. Aggregation operators, which can be t-norms or t-conorms, exist. The first involves operators with the logical connector AND as the minimum operator and those with the connector OR as the maximum operator. The average operator, such as WA (weighted average) and OWA, is another type (ordered weighted average). There are also concentration and dilution operators that take only one input and reduce or increase their membership value. The square of the input value is the simplest concentrator, while the square root of the input value is the simplest dilator. Table 1 summarizes the expressions for the fuzzy operators used in this work, where a and b are their inputs and 0 < r < 1.

**Table 1:** Fuzzy operators

Operator	Expression
Max (a,b)	–
Min (a,b)	–
WA (a,b,r)	r x a + (1 − r) x b
OWA (a,b,r)	r x max (a, b) + (1 − r) x min (a, b)
Dilator (a)	√a
Concentrator (a)	a^2^

**Fig. 3:**
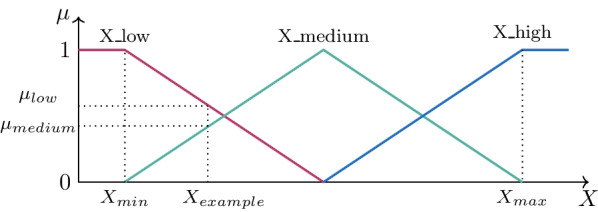
Fuzzification scheme

Fuzzy logic is used to build more meaningful trees in order to improve the interpretability of the evolved models. To that end, we adopted the most straightforward fuzzification scheme presented in Fig. 3, where X is any feature. X_max is the highest X value in the dataset, and X_min is the lowest. The membership functions are triangular, and there are three fuzzy sets for X, which are set as shown in Table 2.

**Table 2:** Linguistic terms used for input X

Fuzzy Sets	Interval
X_low	[X_min, (X_max + X_min)/2]
X_medium	[X_min, X_max]
X_high	[(X_max + X_min)/2, X_max]

**Fig. 4:**
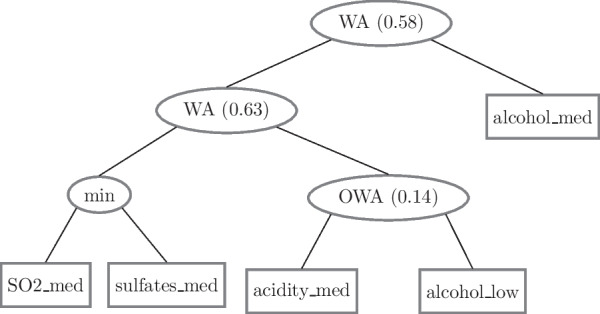
FPT example

Figure 4 shows an FPT example where the tree represents the class "High Quality wine." The alcohol content, acidity, and concentrations of sulfur dioxide and sulfates are the input attributes. They are associated with a fuzzy term that represents a range in the discourse attribute universe. In Fig. 4, for example, the fuzzy term *Alcohol_Low* represents the fuzzy set that indicates a low alcohol content. In fuzzy sets, the membership value is grouped by operators who keep the partial results in the range [0,1]. If the given attributes presented at the bottom of the tree accurately represent the class, the value obtained in the output after all feature groupings must be close to 1.

In our previous research [13, 21–25] we have described and experimented with a wide diversity of algorithms such as K-Nearest Neighbors (KNN) [36], Support Vector Machine (SVM) [37], AdaBoost [38], Random Forest (RF) [39], Light Gradient Boosting Machine (LGBM) [40], Extreme Gradient Boosting (XGB) [41], and Logistic Regressor (LR) [42]. Here, we compared the results obtained by these algorithms with the ones achieved by classifiers synthesized by Decision Trees (DT), GP and GE to check if the results of the interpretable classifiers are competitive.

In addition, the fuzzification scheme employed in the FPTs is also employed as a feature engineering step to generate another representation of the original attributes (FOT parameters). The main motivation to perform the fuzzification is to verify if the fuzzy terms can emphasize the differences between the groups. Besides, the newly generated features can also be used to train the algorithms from previous works to check if it is possible to improve the diagnostic accuracy.

### Performance analysis

In medical diagnosis, the area under the receiver operating characteristics curve (AUC) can measure a model's ability to discriminate whether a condition is present or not, so it is an appropriate metric for this work [43]. Generalization is what makes learning worthwhile. To assess the generalization capacity, we must test a classifier in a different set from the one used for its training. Usually, we desire to use as much data as possible to train the model and the most considerable amount available to test its generalizability However, because our dataset is small, we must use a practical approach, such as the k-fold cross-validation technique [44], to estimate generalization performance and perform hyperparameter tuning. Unfortunately, because the performance estimate was directly optimized while tuning the hyperparameters, using single k-fold cross-validation to complete both tasks may introduce an optimistic bias into the performance estimate. As a result, in our experimental approach, we employ Nested Cross-Validation. This procedure uses an outer cross-validation process to generate a performance estimate that is used to select the best model. To minimize an inner cross-validation estimate of generalization performance, the model’s hyper-parameters are tweaked independently in each fold of the outer cross-validation. The outer cross-validation is simply measuring the performance of a method for fitting a model. As the test data in each iteration of the outer cross-validation has not been used to optimize the performance of the model in any manner, this avoids the bias produced by the flat cross-validation technique and may thus provide a more trustworthy criterion for selecting the best model.

Thus, we divided the dataset into ten folds with the same proportion of classes, enabling ten sub-experiments, each using nine folds for training and one for testing. All algorithms use the same training and test sets so that we can compare their results. In the beginning, we specify some options of hyper-parameters for a specific algorithm. An exhaustive search is made using the inner cross-validation to find the best hyper-parameters in each sub-experiment, which we apply to the respective test fold. After repeating that ten times, we take all test sets' results, make a single ROC curve, and take the AUC for that algorithm.

## Experimental scheme

We performed three experiments, each considering two distinct analyses with the dataset: Control group versus individuals with sarcoidosis and normal spirometry, and control group versus individuals with sarcoidosis and altered spirometry.

Experiment 1 consisted of assessing each FOT feature's ability to diagnose correctly respiratory changes associated with sarcoidosis.

In the second experiment, we evaluated the accuracy of several classifiers in the diagnosis. We also evaluated interpretable methods and other ML algorithms to compare their results. We investigated all techniques using the original dataset with the z-score normalization and a fuzzy dataset with the fuzzification scheme from Fig. 3. For each experiment, we normalized the data considering only the training set, and then the test set is normalized following the same scale. Regarding the fuzzification scheme, we took the minimum and the maximum values for each attribute from the training set, and then when fuzzifying the test set, if there is a lower or a higher value than these limits, they are set up to 0 or 1, respectively.

We implemented KNN, SVM, AdaBoost (using Decision Trees as base estimator), RF, LGBM, XGB, LR, and DT classifiers with the library Scikit-Learn [45]. We can do a grid search to find a model's best hyperparameters with a function from this library. The options provided for the search are in Table 3.

**Table 3:** Hyperparameters for grid search

Classifier	Hyperparameters for tuning	Options
KNN	Number of neighbors	1, 3, 5, 7, 9, 11, 13
SVM	Regularization parameter	1, 2, 5, 7, 10, 50, 100, 200, 400
	Kernel coefficient	0.001, 0.01, 0.05, 0.1, 1
AdaBoost	Number of base estimators	10, 30, 60, 100, 200, 400
	Max depth of base estimators	1, 2, 3, 4, 5, 10, 15, 30, 60
RF	Number of estimators	10, 30, 60, 100, 200, 400
	Max depth of estimators	1, 2, 3, 4, 5, 10, 15, 30, 60
LGBM	Number of estimators	1, 2, 3, 4, 5, 10, 15, 30, 60
	Max depth of estimators	10, 30, 60, 100, 200, 400
XGB	Number of estimators	1, 2, 3, 4, 5, 10, 15, 30, 60
	Max depth of estimators	10, 30, 60, 100, 200, 400
LR	Regularization parameter	0.001, 0.01, 0.1, 1, 10, 100, 1000
DT	Max depth	2, 3, 4, 5, 10, 50
	Criterion	‘gini’, ‘entropy’, ‘log_loss’
	Splitter	‘best’, ‘random’
GP	Population size	100, 300, 500, 1000, 3000
	Number of generations	20, 50, 100, 200
	Initial depth	(2–2), (2–6)
	Tournament size	2, 7, 20
GE	Population size	100, 300, 500, 1000, 3000
	Number of generations	50, 100, 200

We performed GP classifiers with the library gplearn 0.4.1, which is compatible with Scikit-learn; we can do a grid search with the previously mentioned function. Finally, we used ponyGE2 0.2.0 to carry out GE classifiers, but that library is not compatible with Scikit-learn. Because of that, we developed a new interface that allows us to use Scikit-learn functions [46]. Table 3 also shows the options provided to GP and GE hyperparameters.

**Fig. 5:**
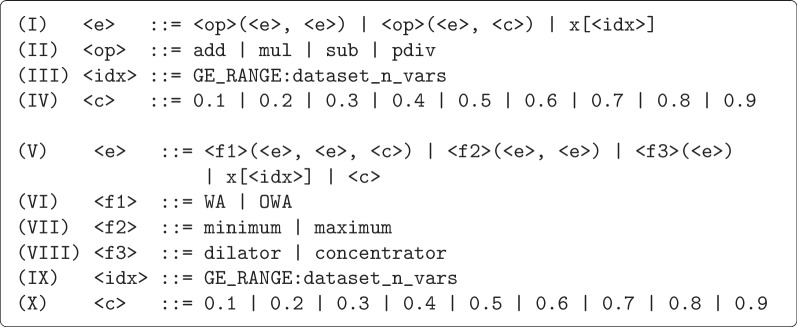
Grammar used by GE

We used arithmetic functions when performing GP with normalized data. In this case, the model's output results from the tree transformed through a sigmoid function. When performing it with fuzzy data, we used the functions shown in Table 1, and the output of the model is directly the result of the tree. Finally, we defined the grammar shown in Fig. 5 for the use of GE, in which rules (I)–(IV) are used in experiments with normalized data and rules (V)–(X) in those with fuzzy data.

Thirdly, we included a feature selection technique and rerun every procedure of experiment 2. We used a recursive feature elimination to select the optimal subset of features. It is a backward method, in which the search starts with all features, eliminating at each iteration the one whose removal presents the most negligible loss of information. We put the same hyperparameters in the grid in Table 3 and another one, which is the number of features to select. There are 16 FOT indexes in total, so we put options 1–15 for that hyperparameter, except in GP and GE experiments. For these, we put only three alternatives (4, 8, 12) due to their execution time. In experiments with fuzzy data, there are 48 features, so we put options 1–47, except in GP and GE experiments, in which there are just three alternatives (12, 24, 36) again.

Employing feature selection to obtain a subset of the optimal features contributes to avoiding overfitting, especially in works with a small dataset like ours. Since reducing the number of features simplifies the model, our principal interest in feature selection is to achieve a better performance in the classification. However, experiment 3 can also contribute to explaining the results by observing which features are selected most often. Each experiment consists of ten sub-experiments. As we use nine algorithms, each analysis shows 90 results in the feature selection. From these results, we can know which are the essential features. We elaborated 3D plots with the three most frequent ones in each analysis to evaluate the visual separation between classes.

We disclosed our code as well as its respective results on https://github.com/danozu/sarcoidosis. All experiments were performed using a random seed equal to 7, which means that their results can be easily reproduced.

### Statistics

Initially, the sample distribution characteristics were assessed using Shapiro–Wilk's test. Since data were non-normally distributed, non-parametric analyses (Mann–Whitney test) were performed. Differences with p ≤ 0.05 were considered statistically significant. These analyses were performed using R version 4.0.5 (R Foundation for Statistical Computing, Vienna, Austria).

## Results

The studied subjects' biometric and spirometric characteristics are described in Table 4. With the exception of the height, the demographics show no significant differences, which decreases the potential confounding by demographics.

**Table 4:** Demographic and spirometric characteristics of the studied subjects

	Control (n = 25)	Sarcoidosis and normal spirometry (n = 24)	Sarcoidosis and altered spirometry (n = 23)	ANOVA p
Age (years)	59.1 ± 10.5	48.6 ± 11.2	47.8 ± 11.2	ns
Body mass (kg)	67.6 ± 15.1	68.2 ± 13.2	73.4 ± 15.5	ns
Height (m)	1.6 ± 0.1	1.6 ± 0.1	1.7 ± 0.1	0.019
BMI (kg/m^2^)	26.7 ± 5.0	26.8 ± 5.2	26.3 ± 4.5	ns
Male/Female	6/19	5/19	9/14	–
FVC (L)	3.1 ± 0.9	3.1 ± 0.8	3.2 ± 1.4	ns
FVC (%)	100.2 ± 20.3	99.2 ± 18.1	86.2 ± 28.8	ns
FEV_1_ (L)	2.5 ± 0.7	2.5 ± 0.7	2.2 ± 0.9	ns
FEV_1_ (%)	100.0 ± 21.3	96.4 ± 17.8	74.5 ± 23.8	0.0001
FEV_1_/FVC	80.3 ± 6.5	80.6 ± 6.8	72.0 ± 8.5	0.0001
FEV_1_/FVC (%)	99.8 ± 7.1	97.8 ± 8.3	87.3 ± 9.6	0.0001
FEF_25–75%_ (L)	2.7 ± 1.2	2.9 ± 1.1	1.7 ± 0.7	0.0003
FEF_25–75%_ (%)	110.7 ± 45.9	96.6 ± 44.0	51.0 ± 19.6	0.0001

**Fig. 6:**
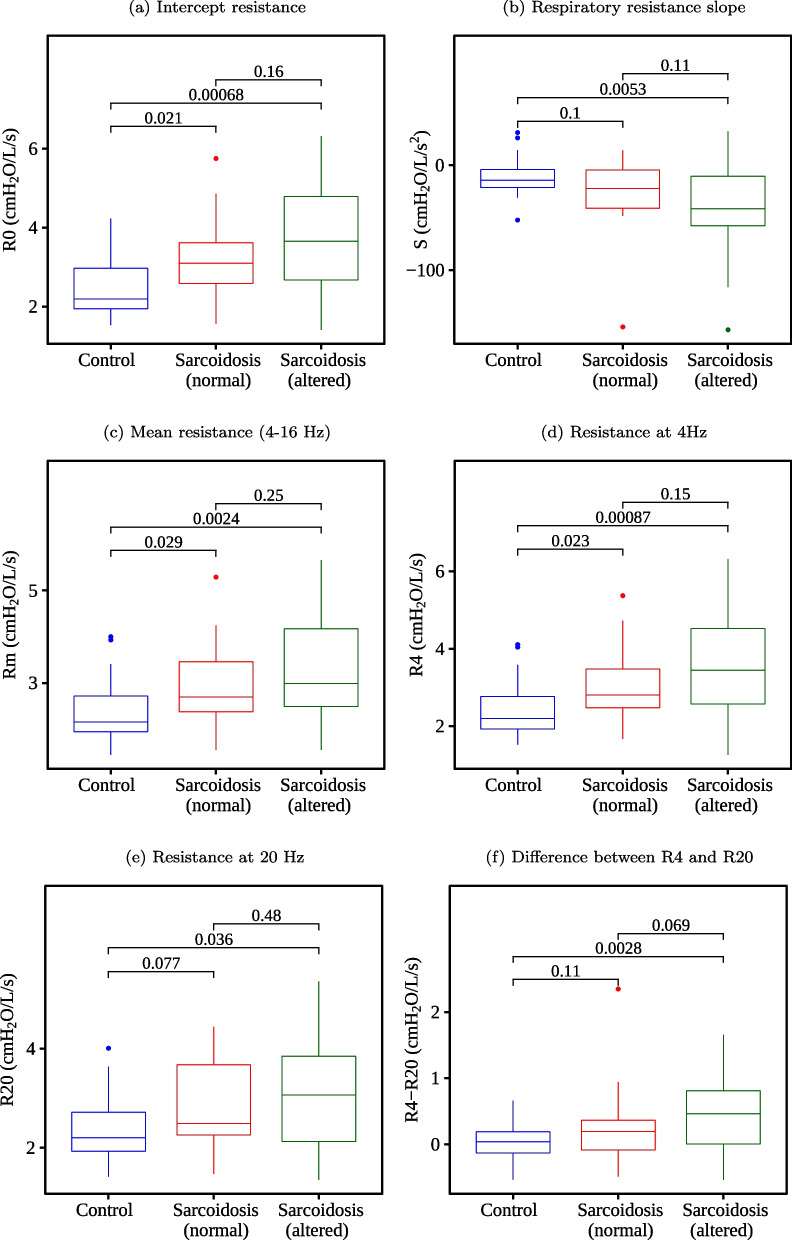
Boxplots of the resistive parameters and their respective p-values

**Fig. 7:**
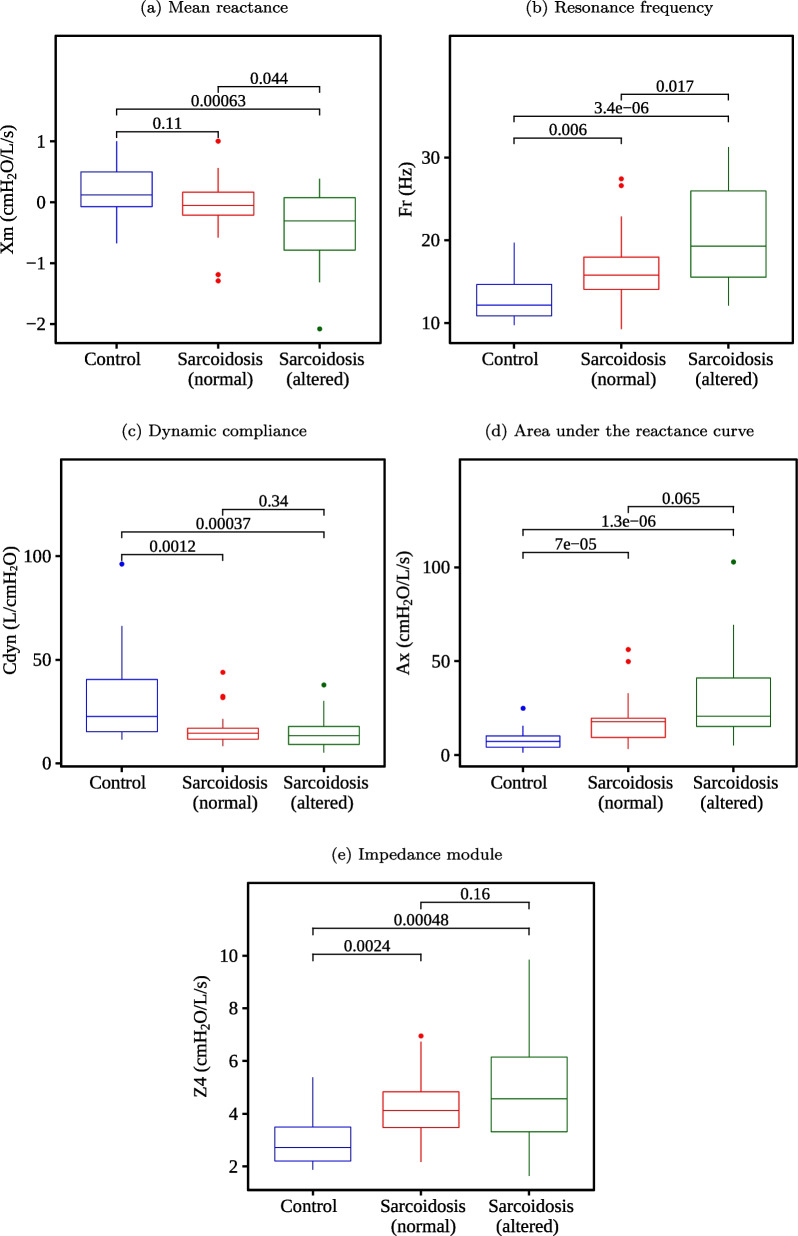
Boxplots of the reactive parameters and their respective p-values

**Fig. 8:**
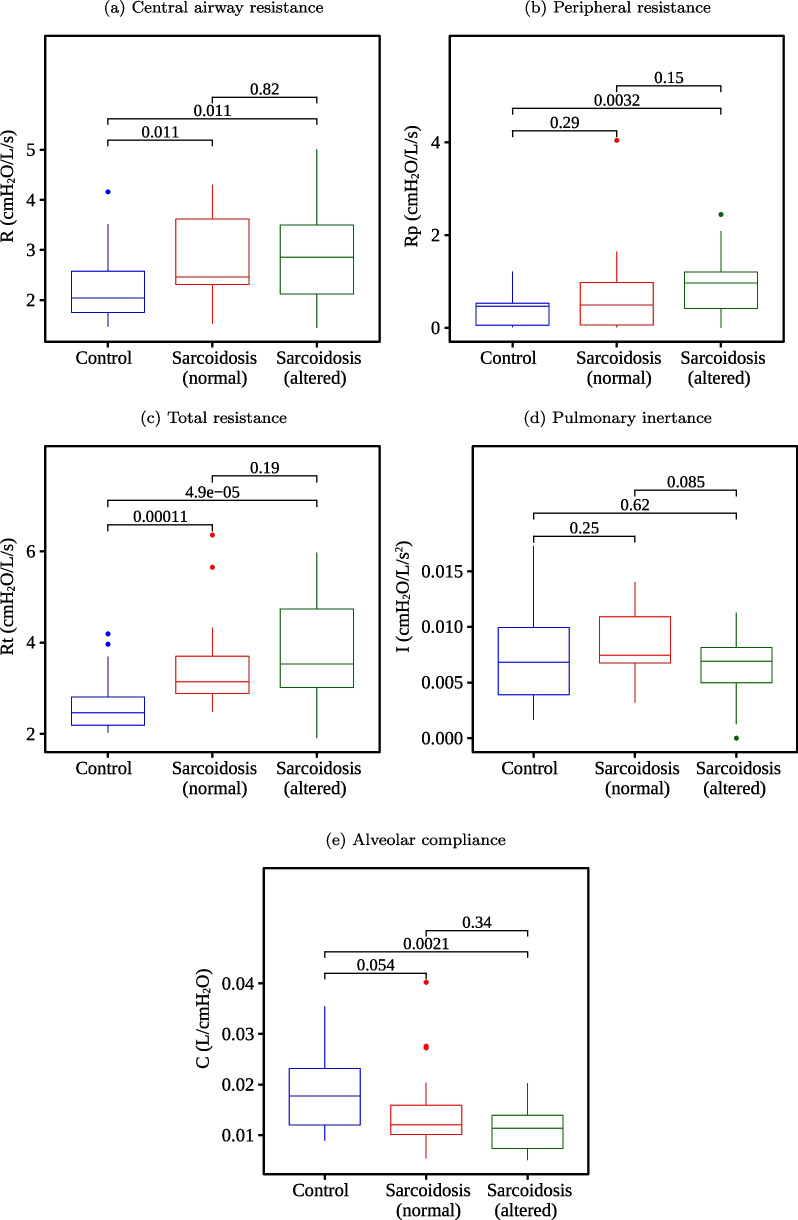
Boxplots of the results obtained using the eRIC model and their respective p-values

Figure 6 shows the boxplots of the resistive features used in this work. A similar analysis for the reactive features is presented in Fig. 7, while Fig. 8 shows the results obtained from the eRIC model.

When comparing the control group with the sarcoidosis and normal spirometry group, we have found no significant changes (p > 0.05) in the features S, R20, R4–R20, Xm, R, I, and C. Otherwise, Ax and Rt presented the best p-values (p < 0.001). When analyzing the control group with sarcoidosis and the altered spirometry group, there were no significant changes (p > 0.05) just in I. While the best p-values (p < 0.001) were found in R0, R4, X_m_, F_r_, C_dyn_, A_x_, Z4, and R_t_. When examining individuals with sarcoidosis, we have found no significant changes (p > 0.05) between groups with normal and altered spirometry in most features, except X_m_ and F_r_.

Fuzzifying data can improve the comparisons described in the previous section with new observations. As the fuzzification scheme in Fig. 3 triples the number of features, since we add the membership values obtained by the feature in each fuzzy set. Therefore, we decided not to present boxplots for fuzzy data. Table 5 shows the fuzzy features that present significant changes (p < 0.05) between groups, while Table 6 presents the quantity of non-zero values in the fuzzy features.

**Table 5:** Fuzzy features with significant changes between groups. Note that Control versu s Sarcoidosis (altered) presents 33 features distributed in two columns

Control versus Sarcoidosis (normal)		Control versus sarcoidosis (altered)			
Feature	p-value	Feature	p-value	Feature	p-value
Ax_low	0.00007	Ax_low	0.0000068	C_high	0.0036
Ax_medium	0.00007	Fr_low	0.000012	Rp_low	0.0039
Rt_low	0.00024	Ax_medium	0.000065	Rm_low	0.0042
Cdyn_low	0.0015	Rt_low	0.00014	S_high	0.006
Rt_medium	0.0023	Fr_high	0.0003	Rm_high	0.0077
Z4_low	0.003	Cdyn_low	0.00056	R4-R20_medium	0.0084
Z4_medium	0.0035	Z4_low	0.00068	Rt_medium	0.009
Fr_low	0.0067	Z4_high	0.00069	Z4_medium	0.011
Cdyn_medium	0.0072	Xm_high	0.0011	Xm_low	0.012
R_low	0.019	R0_low	0.0016	R4_high	0.015
Fr_medium	0.022	R4_low	0.0019	R4-R20_high	0.016
R0_low	0.028	Rt_high	0.0023	I_high	0.016
R4_low	0.028	C_low	0.0025	S_low	0.016
C_low	0.039	R0_high	0.003	R_low	0.017
C_medium	0.049	Cdyn_medium	0.0031	C_medium	0.029
		Rp_medium	0.0032	Fr_medium	0.034
		R4-R20_low	0.0034

**Table 6:** Quantity of non-zero values in the fuzzy features

Feature	Qty	Feature	Qty	Feature	Qty
R0_low	55	R4-R20_medium	70	Z4_high	11
R0_medium	70	R4-R20_high	7	R_low	54
R0_high	17	Xm_low	11	R_medium	70
S_low	6	Xm_medium	70	R_high	19
S_medium	70	Xm_high	61	Rp_low	69
S_high	66	Fr_low	59	Rp_medium	70
Rm_low	55	Fr_medium	70	Rp_high	3
Rm_medium	70	Fr_high	13	Rt_low	58
Rm_high	17	Cdyn_low	67	Rt_medium	70
R4_low	57	Cdyn_medium	70	Rt_high	14
R4_medium	70	Cdyn_high	5	I_low	49
R4_high	15	Ax_low	68	I_medium	70
R20_low	53	Ax_medium	70	I_high	23
R20_medium	70	Ax_high	4	C_low	61
R20_high	19	Z4_low	61	C_medium	70
R4-R20_low	65	Z4_medium	70	C_high	11

**Fig. 9:**
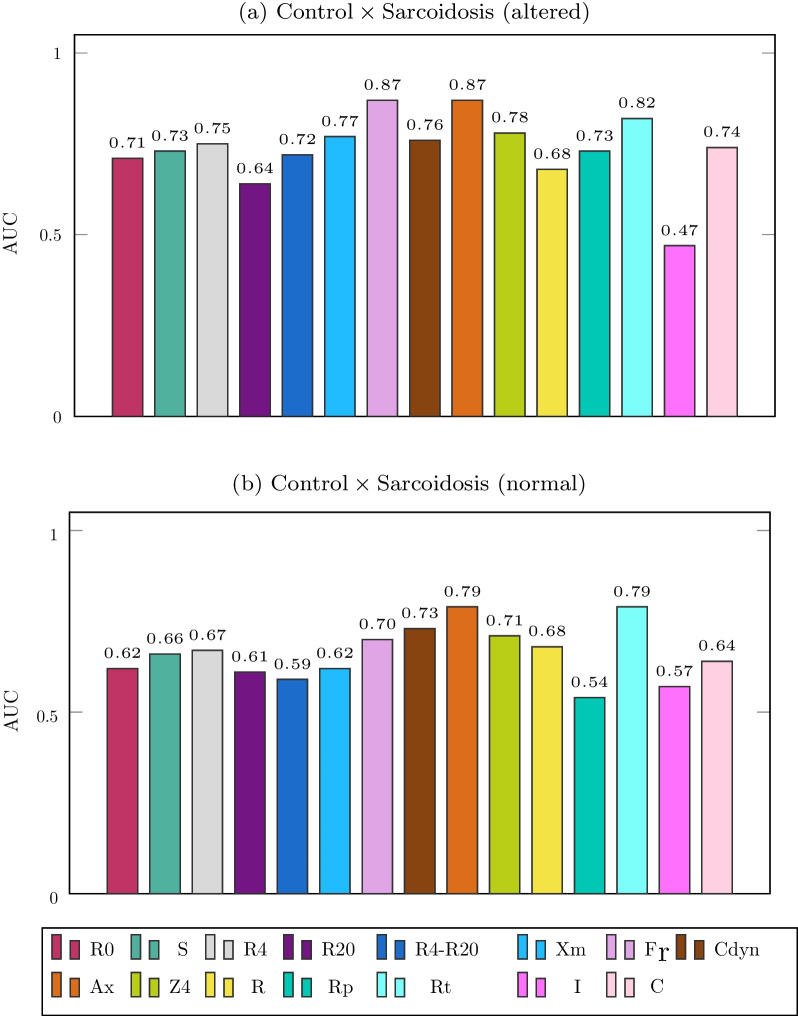
Results of experiment 1

We analyzed each FOT parameter individually to test its performance in the classification of groups. Figure 9 shows the results. In the control group versus sarcoidosis (altered) analysis, the best FOT parameter (BFP) was F_r_, followed by A_x_ and R_t_, which presented AUC equal to 0.87, 0.87, and 0.82. In the analysis with normal spirometry, no feature achieved an AUC greater than 0.80. The BFPs were A_x_ and R_t_, both with AUC equal to 0.79.

**Fig. 10:**
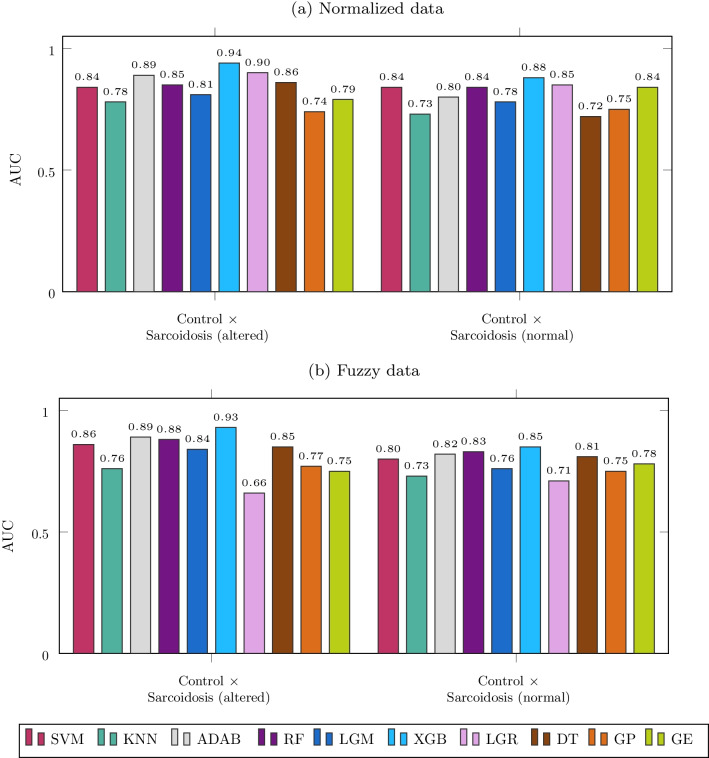
Results of experiment 2

Figure 10 shows the results of experiment 2 with both normalized and fuzzy data. Firstly, using normalized data in the control group vs. sarcoidosis (altered) analysis, the best results were XGB, ADAB, and LGR, which presented AUC equal to 0.94, 0.90, and 0.89, respectively. While in the analysis with normal spirometry, no algorithm achieved an AUC greater than 0.90, the best ones XGB and LGR, presented AUC equal to 0.88 and 0.85.

Next, using fuzzy data, we can see in the control group vs. sarcoidosis (altered) analysis that the best result was with XGB, which presented AUC equal to 0.93, followed by ADAB, with AUC equal to 0.89. In the analysis with normal spirometry, no algorithm presented an AUC greater than 0.85. Again, XGB was the best method, showing AUC equal to 0.85.

**Fig. 11:**
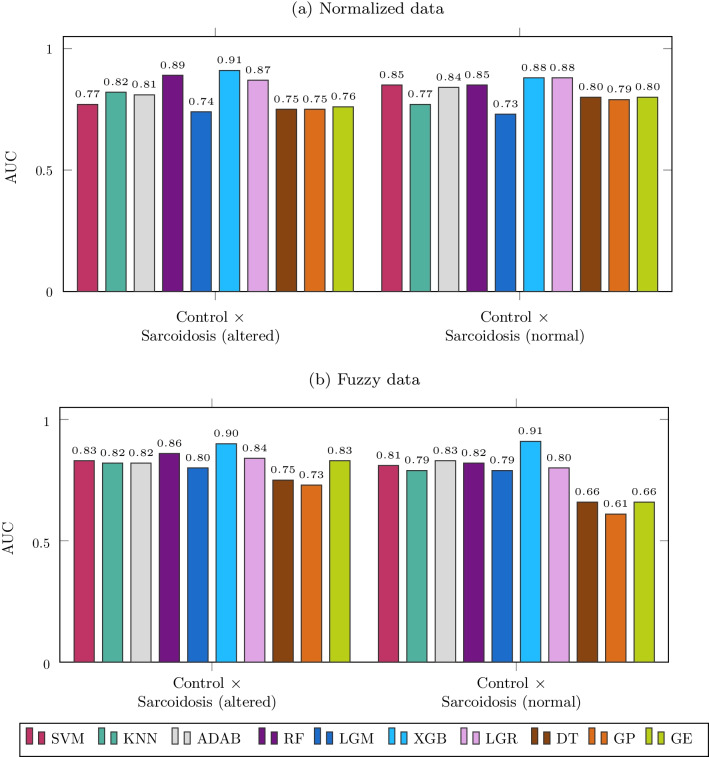
Results of experiment 3

Figure 11 shows the results of experiment 3 with both normalized and fuzzy data. Concerning the performance with all features, the main improvements refer to the KNN performance in the analysis with altered spirometry and RF with normal spirometry, both using fuzzy features.

For experiments 2 and 3, we also provided the ROC curves as supplementary material. These graphs can be found in https://github.com/danozu/sarcoidosis/tree/master/results/ROC_curves.

It is worth mentioning that the first performance in the analysis with normal spirometry shows AUC ≥ 0.90, which we achieved with the XGB using fuzzy features.

We can further explain the results by observing which attributes are selected most frequently. According to our experimental scheme, we did each analysis with ten sub-experiments. Thus, with nine methods and two analyses each, a total of 180 sub-experiments were carried out to present the results with normalized data and the same amount for fuzzy data. Table 7 displays the percentage of selection of each normalized feature in the respective 270 experiments, while Table 8 shows the percentage of selection of the most frequent fuzzy features in each analysis.

**Table 7:** Percentage of selection of normalized features

Feature	Control versus sarcoidosis (normal) (%)	Control versus sarcoidosis (altered) (%)
R0	26	43
S	24	14
Rm	79	54
R4	26	76
R20	20	21
R4–R20	63	74
Xm	76	69
Fr	80	94
Cdyn	84	90
Ax	91	29
Z4	44	31
R	74	36
Rp	49	66
Rt	99	94
I	59	61
C	80	64

**Table 8:** Percentage of selection of fuzzy features

Control versus sarcoidosis (normal spirometry)		Control versus sarcoidosis (altered spirometry)	
Feature	Percentual (%)	Feature	Percentual (%)
Rt_low	89	Fr_low	99
Ax_low	84	I_medium	93
Fr_low	81	Fr_high	86
I_medium	80	C_high	79
I_low	77	I_low	66
C_high	77	Ax_low	63
Rm_low	76	C_medium	49
Rp_medium	69	Ax_medium	43
Rp_low	67	Rp_medium	39
Xm_low	66	R_medium	31
Ax_medium	56	R4-R20_medium	29

**Fig. 12:**
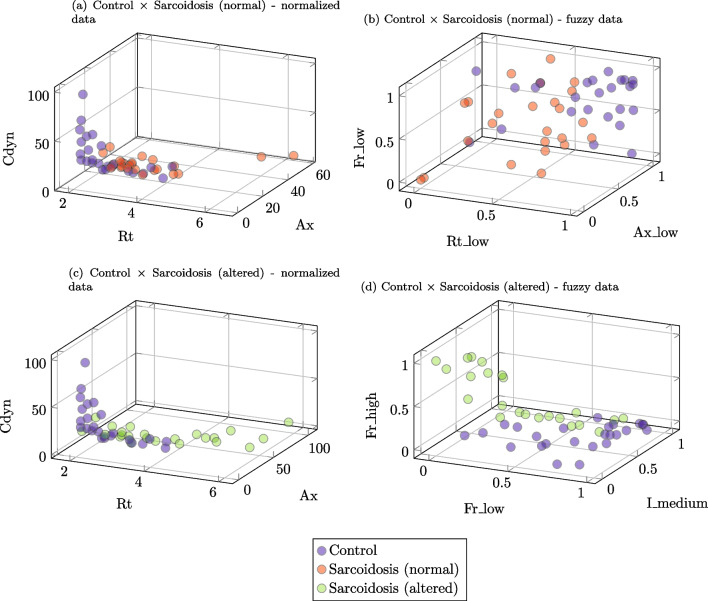
Data representation with the three main features. Control group x sarcoidosis with normal spirometry using normalized (**A**) and fuzzy (**B**) data. Control group x sarcoidosis with abnormal spirometry using normalized (**C**) and fuzzy (**D**) data

In order to develop a visual and intuitive analysis of the differences between the groups, we used the three most frequent ones shown in Tables 7 and 8 to create 3D graphics, as presented in Fig. 12.

To corroborate the results shown in Tables 7 and 8, we picked the weights for each feature in the LR experiments and calculated the average. The features with the most significant averages in absolute values in experiment 2 were A_x_, F_r_, and C_dyn_ for normal spirometry and normalized data; F_r_, A_x_, and C_dyn_ for altered spirometry and normalized data; Xm_low, C_high, and Ax_low for normal spirometry and fuzzy data; and Fr_low, Fr_high and I_medium for altered spirometry and fuzzy data. These are the essential features according to the LR experiments. The results for all features are in https://github.com/danozu/sarcoidosis/tree/master/results/LR_features_weights_average.

**Fig. 13:**
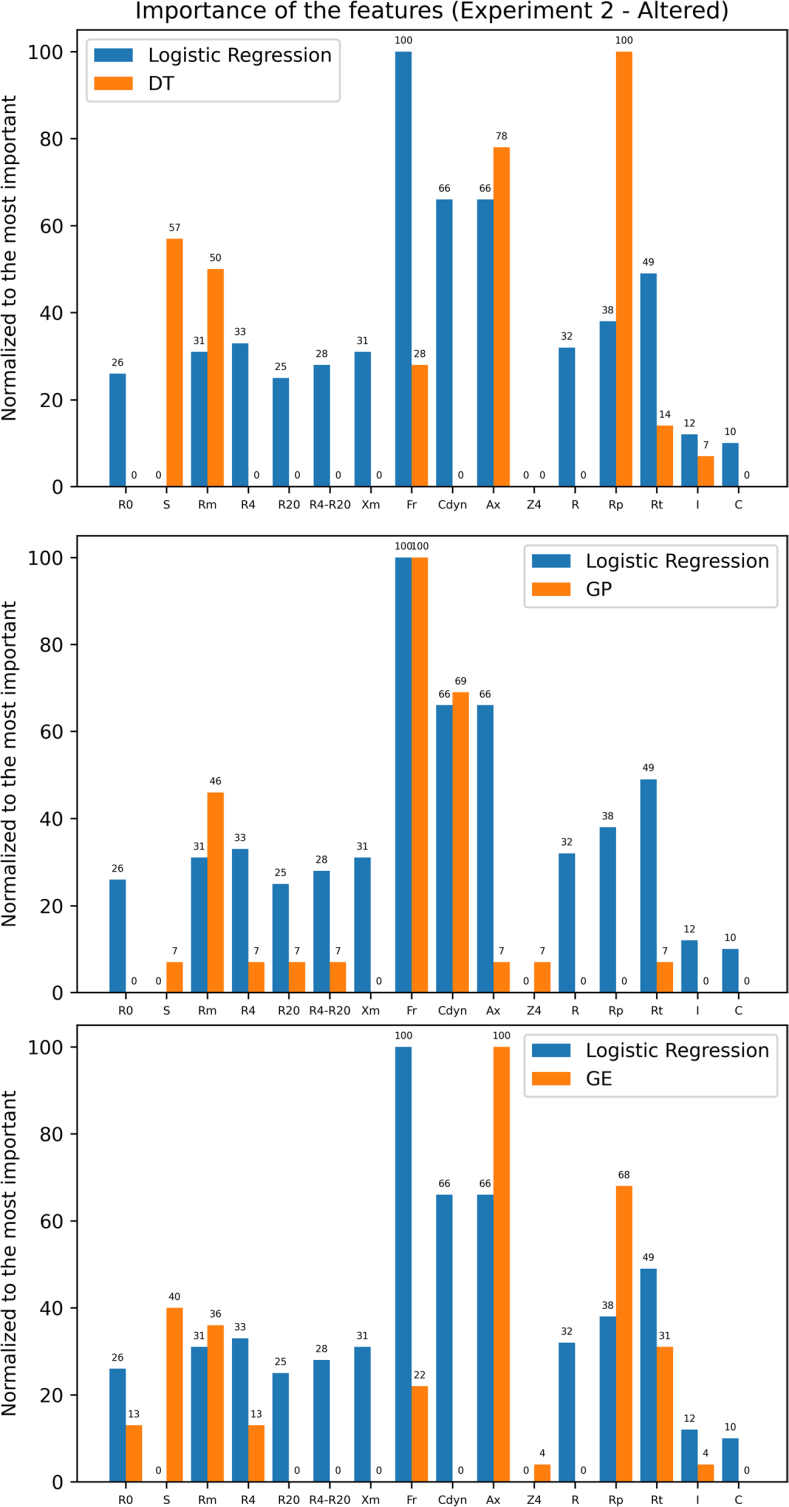
Comparison between the importance of the features according to Logistic Regression, decision trees, genetic programming and grammatical evolution for experiments with altered spirometry

**Fig. 14:**
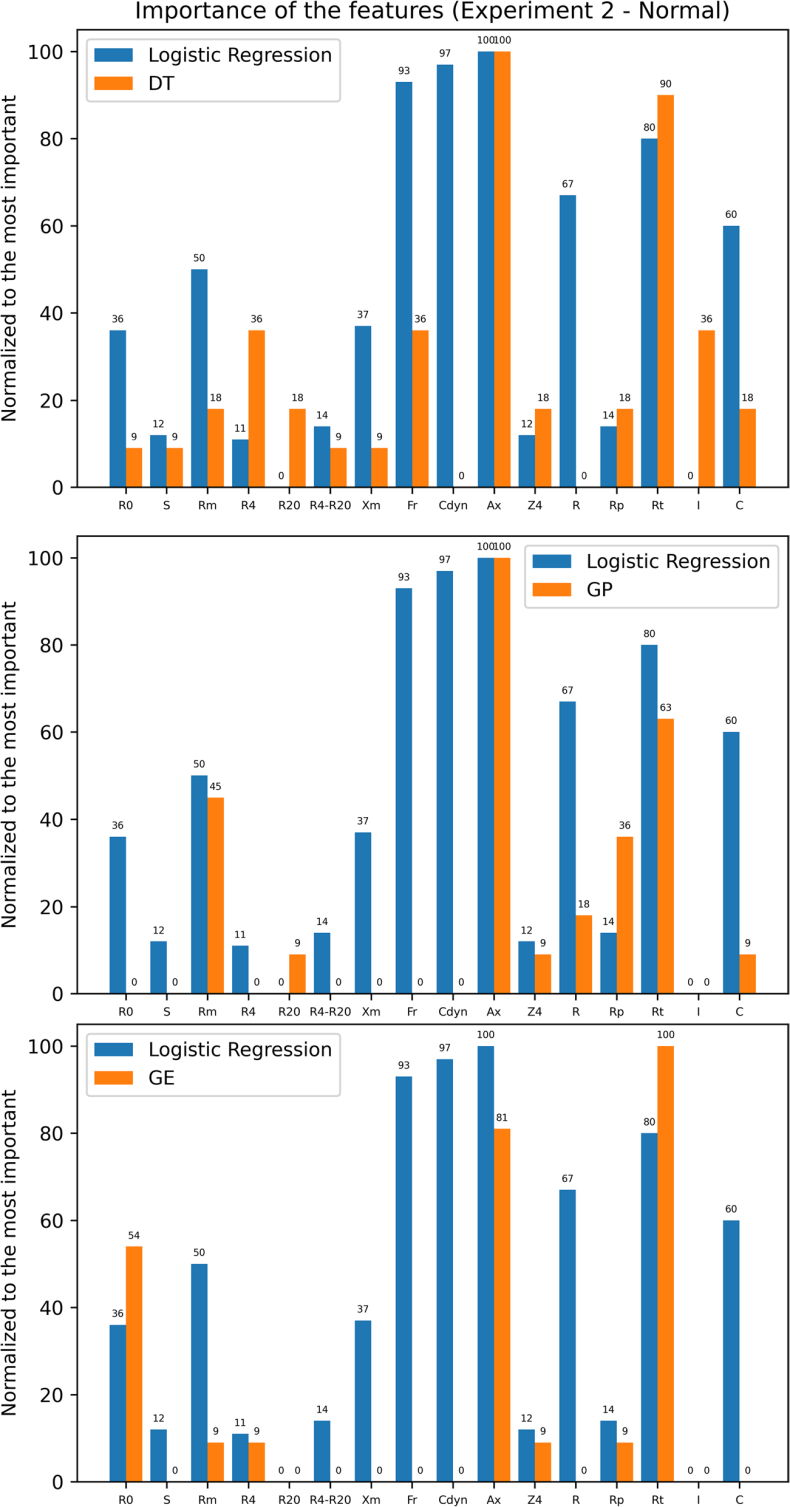
Comparison between the importance of the features according to Logistic Regression, decision trees, genetic programming and grammatical evolution for experiments with normal spirometry

We also made public the expressions generated by interpretable models (DT, GP, and GE) for each experiment in https://github.com/danozu/sarcoidosis/tree/master/results/Interpretable_expressions. Then, we carried out a comparison between the results from the LR experiments with these results. For this, we normalized between the minimum and maximum of the absolute values found in the LR experiments and normalized the number of times each feature was used in the expressions found as final solutions in the DT, GP, and GE experiments. This comparison is presented in Figs. 13 and 14.

From Fig. 10, we know that for normalized data, the interpretable model with the best accuracy was DT for experiments with altered spirometry and GE for experiments with normal spirometry. However, in both cases, the experiments with LR presented better accuracy. Analyzing Fig. 13, the most significant differences between LR and DT results are related to frequent use of F_r_, C_dyn_, and R_t_ by LR, while DT presented a more frequent use of S and R_p_. At the same time, analyzing Fig. 14 to compare LR to GE, the most significant differences are related to more frequent use of F_r_, C_dyn_, R, and C by LR.

## Discussion

To the best of our knowledge, this is the first study to develop ML classifiers to assist in the diagnosis of respiratory changes associated with sarcoidosis based on FOT exams. The results showed that automatic classifiers could increase sarcoidosis diagnosis accuracy, especially in individuals with normal spirometry. Genetic Programming and Grammatical Evolution were particularly beneficial because they provide intelligible expressions to make the classification.

The three studied groups were of comparable age, weight, and BMI, showing only small differences in height (Table 4). The modifications in spirometric parameters were consistent with previous studies [3, 47, 48] showing reduced values in sarcoidosis.

Respiratory changes observed in Figs. 4 and 5 were consistent with previous studies from our group [49] and studies using impulse oscillation systems (IOS) to evaluate the association of respiratory impedance, pulmonary function, and airway wall thickness [45]. They were also in line with the use of IOS to evaluate lung capacity deterioration in sarcoidosis [46].

The results presented in Table 5 demonstrate that fuzzification can contribute to the explanation of the results by looking at the importance of a feature, observing how many times the models choose that particular feature. For example, we realized that Fr is relevant in diagnosing sarcoidosis, both with normal and altered spirometry. We learned that the fuzzy feature Fr_high is important in the analysis with altered spirometry and not with the normal one with fuzzification. Intrinsically, some piece of information in that term is perceived, i.e., there are few individuals with high F_r_ in the control and normal spirometry groups. Indeed, we can see that its highest values are concentrated in the group with altered spirometry when analyzing the F_r_ boxplot in Fig. 7. Also, we can observe in Table 8 that this fuzzy feature is chosen by the feature selection mechanisms 86% of the time, which indicates that it is indeed an important feature. We can do the same observation regarding R_t_, which is relevant in diagnosing sarcoidosis, both with normal and altered spirometry, but R_t__high is not relevant in the analysis with normal spirometry.

Another observation is that several fuzzy features have a lower p-value when compared to their respective FOT features, denoting greater significant changes between the groups. For example, between control and sarcoidosis (altered) groups, I presented p = 0.62, being the worst feature, while high_I presented p = 0.016 in the same analysis. Likewise, C presented p = 0.054 in the analysis with normal spirometry, while C_low and C_medium presented, respectively, p = 0.039 and p = 0.049. We can understand that a concentration of relevant information in certain fuzzy features reduces or eliminates outliers' influence. In this case, the fuzzy features C_low and C_medium provide a better description of the values of the FOT feature C. In this way, we can also understand why there are almost no features with their respective three fuzzy features in Table 5. There is none in the analysis with normal spirometry, and in the analysis with altered spirometry, there are only five (F_r_, R_t_, Z4, C, and R4–R20).

The quantity of non-zero values in the fuzzy features described in Table 6 can be interpreted as measuring the amount of information present in each fuzzy feature. According to the fuzzification scheme presented in Fig. 3 which describes the fuzzification of a feature call X, we were already expecting that X_medium had 70 non-zero values. There are 72 samples in our dataset, and only the highest and the lowest values of each feature have no membership value in X_medium, presenting value 1 in X_high and X_low. Additionally, if a feature X were equally distributed, above and below its average, so X_low and X_high would have each one around 36 non-zero values. However, no feature comes close to this quantity due to their irregular data distribution, as seen in the boxplots. In extreme cases, notably R, A_x_, C_dyn_, S, and R4–R20, we can explain it by their respective boxplots, in which these features are the ones that have the outliers more distant from the mean values. For example, in S, this leads to a S_min much smaller than the S's average, and therefore few samples will have membership value in S_low. In the other mentioned cases, outliers much higher than the features' average lead to a too high X_max, allowing few samples with membership values in X_high. The same occurs, to a lesser extent, with other features, so that many fuzzy features may be irrelevant. While this may appear to be a problem, it can be helpful in models with a feature selection step or methods with an embedded feature selection, such as the classifiers synthesized by GP or GE. When fuzzifying a feature, it is true that it projects the data in a higher dimension space since one feature is now represented by three features in this new space. However, if one can be irrelevant, the other two's quality can be even higher than that of the original one since they have less information from outliers. For example, the Ax's highest outlier has a membership value of 1 in Ax_high and 0 in the others, while the rest of the samples have their information concentrated in Ax_low and Ax_medium. Even the other three samples, which have a non-zero membership value in Ax_high, also have a value in Ax_medium, influencing the results even if Ax_high is discarded.

Indeed, in terms of the p-values, we can observe extremely low p-values in some features. Ax stood out in the control group vs. sarcoidosis (normal) analysis (Fig. 7D p = 0.00007) and in the control group vs. sarcoidosis (altered) analysis (p = 0.0000013). When fuzzifying, it continued to stand out in both analyses with Ax_low (p = 0.00007 and p = 0.0000068, respectively) and Ax_medium (p = 0.00007 and p = 0.000065, respectively), while Ax_high did not present significant changes between the groups (p = 0.33 and p = 0.069, respectively), being one of the worst features in this assessment. It indicates that the fuzzy features better represent the range of values that are useful for class discrimination. The same observations can be made with R_t_ (p = 0.00011 and p = 0.000049, respectively), becoming R_t__low (p = 0.00024 and p = 0.00014, respectively), and with Cdyn (p = 0.0012 and p = 0.00037, respectively), becoming Cdyn_low (p = 0.0015 and p = 0.00056, respectively), among others.

In the first experiment (Fig. 9), we analyzed each FOT parameter individually to test its performance to distinguish between groups. When identifying patients with sarcoidosis and altered spirometry, the best FOT parameters (BFPs) were F_r_ and _Ax_, which presented AUC equal to 0.87. The BFPs were A_x_ and R_t_ in the normal spirometry cases, both with AUC equal to 0.79. These results agree with Figs. 4, 5, 6, in which these features obtained the most significant changes in the cited comparisons. These results contrast with previous analysis suggesting that the best feature to identify respiratory changes associated with sarcoidosis in individuals with altered spirometry was Z4, followed by R0 and Rm, while in individuals with normal spirometry, the best feature was R0 [49]. It is worth mentioning that this previous work did not analyze the eRIC model. In the present work, we included other features; some of them have shown promise, especially Ax and Rt.

In the second experiment (Fig. 10), we used automatic classifiers to check if they could improve accuracy over BFPs. We observed that XGB, AdaBoost, and LGR achieved a higher accuracy with altered spirometry and normalized data. The same occurred with XGB, RF and AdaBoost when using fuzzy data. We verified that XGB, AdaBoost, LGR, GE, RF, and SVM achieved a higher accuracy with normal spirometry and normalized data. The same occurred with XGB, RF, AdaBoost, SVM, and LGR when using fuzzy data. Data in Fig. 9 demonstrates that many automatic classifiers incremented the accuracy of sarcoidosis diagnosis. Some of them achieved high accuracy (AUC > 0.90).

In addition to correctly supporting a diagnosis achieving high accuracy, GP, an interpretable method, can also help to understand a bit more about sarcoidosis from intelligible expressions. For example, the expression WA (Fr_medium, Fr_high, I_medium) is an actual final GP individual from our experiments with fuzzy data, which achieved AUC equal to 0.94 in identifying patients with altered spirometry. That expression means the average between Fr_medium and Fr_high, weighted by I_medium. Thus, if I_medium is greater than 0.5, Fr_medium has more influence on the result, else Fr_high is more influential.

An example from our experiments using GE to identify patients with normal spirometry is add(mul(A_x_, 0.7), sub(Rt, Rm)), which achieved AUC equal to 0.84. That expression means (0.7 × A_x_ + R_t_ − R_m_), which is easy to understand. Regarding our experiments with FPTs, an example of solution is OWA (concentrator(Rp_medium), Ax_medium, 0.6), which is the same as 0.6 × max(Rp_medium^2^, Ax_medium) + 0.4 × min(Rp_medium^2^, Ax_medium). Most of the final individuals in our experiments using GP or GE are tiny because our experimental scheme is directed to achieve generalization. Since we have a small dataset, the GP or GE individuals must be simple individuals to reach generalization. This is a crucial point since sarcoidosis is a rare disease. Hence, it was tough to collect this small dataset. It is important to note that the explainable models achieve similar performance. LR is a less complex model, so that it can deal with a small number of samples. In the other explainable methods, we restricted the size of the trees to generate less complex models to avoid the overfit that might make it difficult to generalize the results. As in the comparisons between groups with FOT features, the number of features with significant changes (p < 0.05, Table 5) is much higher between the control and sarcoidosis groups with altered spirometry. We were already expecting more evident respiratory changes between these groups. When comparing the control group with the sarcoidosis and normal spirometry group, we have found significant changes (p < 0.05) in 15 features, and Ax_low, Ax_medium, and Rt_low presented the best p-values (p < 0.001). It is worth noting that significant changes were observed in R_low, C_low, and C_medium, changes which were not observed in the respective original features. It is important to note that when we are dealing with the fuzzy features, we are actually comparing the membership values that the features has in each fuzzy. This membership values are numbers that go from 0 to 1 and therefore they can be compared to obtain the p-values.

When analyzing the control group with sarcoidosis and altered spirometry group, there were significant changes (p < 0.05) in 33 features (Table 5), and Ax_low, Fr_low, Ax_medium, Rt_low, Fr_high, Cdyn_low, Z4_low, Z4_high presented the best p-values (p < 0.001). Likewise, it is worth noting that significant changes were observed in I_high, while the same did not occur when we analyzed I.

In the third experiment (Fig. 10), we used a feature selection technique to verify if it could improve accuracy over BFPs and bring more interpretability. We verified that XGB and RF achieved higher accuracy in conditions of altered spirometry and normalized data. The same occurred with RF, AdaBoost, and XGB when using fuzzy data. We verified that XGB, AdaBoost, LGR, GE, RF, and SVM achieved a higher accuracy with normal spirometry and normalized data. The same occurred with RF, SVM, XGB, and LGR when using fuzzy data. These results demonstrate that the feature selection incremented the accuracy in sarcoidosis diagnosis with several methods. Some of them achieved high accuracy (AUC > 0.90), including in the analysis in patients with normal spirometry, which happened twice.

From Table 7, we observed in control vs. sarcoidosis (normal) that the most frequent features were R_t_, A_x_, and C_dyn_, which were precisely the three best in the individual experiment (Fig. 9). Likewise, in control vs. sarcoidosis (altered) analysis, the most frequent ones were R_t_, F_r_, and C_dyn_, which are also among the best ones in the individual experiment (Fig. 9). However, Ax was the best in the individual one, and in this one was selected a few times. Although relevant individually, we can assume that a specific feature can contribute little when combined with others. On the contrary, I was the worst in the two analyses and appeared far above the least frequent in this experiment. It is worth noting that the number of features selected in each experiment is a hyperparameter included in the grid search, which varies from 1 to 15 in experiments with normalized data and 1–47 in those with fuzzy data, except in the cases of GP and GE, due execution time. We tried with 4, 8, or 12 normalized features and 12, 18, or 24 fuzzy features in these experiments. Thus, without pre-establishing the number of features, they are not selected beyond the minimum necessary. Therefore, observing the frequency of a particular feature in the experiments becomes something more relevant to the results' explanation. It is interesting to note that the backward method can eliminate an essential feature at the beginning when the contribution of each one to the performance is low.

From Table 8, we observed in control vs. sarcoidosis (normal) analysis that the most frequent features were Rt_low and Ax _low, which in Table 5 were among those with best p-values (p < 0.001), followed by Fr_low, which also had significant changes (p < 0.01). In control vs. sarcoidosis (altered) analysis, Fr_low, I_medium, and Fr_high were the most frequent. The first and the third ones were among those with the best p-values (p < 0.001) in Table 5, while the second one did not even show significant changes (p > 0.05). As in the previous analysis, we can assume that a specific feature can contribute much when combined with others, even though it is weak individually. The results when using fuzzification were, in general, close in comparison to experiments in which it was not used. However, there is a contribution to the explanation of the results, because as seen, the fuzzy terms bring more information intrinsically.

The use of the three most frequent parameters shown in Tables 7 and 8 to create 3D graphics is presented in Fig. 12. As can be seen, it is hard to design a simple separation surface in conditions of normal spirometry (Fig. 12A, B). This discrimination is more straightforward in patients with altered spirometry (Fig. 12C, D). In this case, most of the data is slightly separated into different classes, especially using fuzzy data (Fig. 12D).

The best performances rose from 0.79 (Fig. 9B) to 0.91 (Fig. 11B) in normal spirometry and from 0.87 (Fig. 9A) to 0.94 (Fig. 10A) in the analysis with altered spirometry. Compared with previously published studies, this represents an improvement in AUC similar to that previously observed using automatic classifiers in other diseases and conditions performed by our group [22, 24, 25]. They were also similar to previous works of other researchers describing improvements in the diagnostic accuracy of respiratory exams based on magnetic resonance [50], spirometry [51], and pulmonary sounds [52].

Previous research has established that diagnostic easiness is a fundamental attribute for occupied non-specialist clinicians [53]. Studies in radiology [54], ophthalmology [55], and cardiology [56] have shown that ML methods may contribute to improving the medical service by AI-assisted workflow. The present study confirms and extends these findings to respiratory physiology showing that machine learning algorithms help diagnose respiratory abnormalities in sarcoidosis. That is especially true in patients with normal spirometry because the identification is more complicated, without any feature in the individual experiment reaching an AUC of 0.80. In addition, of the exploration of the importance of features in the several experiments can contribute to identification of the more discriminative features to identify patients with sarcoidosis and to contribute to better comprehension of the disease.

## Conclusion

A clinical decision support system for the automatic diagnosis of respiratory abnormalities in patients with sarcoidosis was developed in the present study. This was the first study to propose such a system and evaluate its performance in sarcoidosis.

The best results for each attribute in the classification of the groups achieved only moderate accuracy in normal and altered spirometry. In close agreement with previous results, the use of ML methods resulted in increased performance, resulting in high diagnostic accuracy in patients with normal and abnormal spirometric exams.

The proposed system promises to provide decision support for clinicians when they are struggling to give a confirmed clinical diagnosis. Clinicians may reference the prediction results and make better decisions, improving the productivity of pulmonary function services by ML-assisted workflow.

## Data Availability

The datasets used and/or analysed during the current study are available from the corresponding author on reasonable request.
